# A one health-focused literature review on bovine and zoonotic tuberculosis in Pakistan from the past two decades: challenges and way forward for control

**DOI:** 10.1016/j.onehlt.2024.100763

**Published:** 2024-05-23

**Authors:** Zahid Fareed, Aysha Rana, Syeda Anum Hadi, Annemieke Geluk, Jayne C. Hope, Hamza Khalid

**Affiliations:** a Veterinary Research Institute*,* Lahore*,* Punjab*,* Pakistan; b Consultant-Technical Coordinator, Health Security Partners*,* Islamabad*,* Pakistan; c Department of Infectious Diseases*,* Leiden University Medical Center*,* Leiden*,* the Netherlands; d Division of Immunology*,* The Roslin Institute*,* University of Edinburgh*,* EH25 9RG*,* UK; e Center for Inflammation Research, The Queen's Medical Research Institute, Edinburgh BioQuarter, 47 Little France Crescent*,* Edinburgh EH16 4TJ*,* UK

**Keywords:** Bovine tuberculosis (bTB), *Mycobacterium bovis* (*M. bovis*), Pakistan, Public health, Zoonotic tuberculosis (zTB)

## Abstract

Bovine tuberculosis (bTB), caused by *Mycobacterium bovis* (*M. bovis*), is a globally prevalent zoonotic infectious disease. World Organization for Animal Health (WOAH) estimates indicate that up to 10% of the total human TB cases in developing countries are attributed to *M. bovis*. Pakistan ranks 4th in global milk production with a livestock population of over 212 million animals. Over 8 million families are involved in raising these animals as a means of livelihood. To date, there is an absence of national-level data on the prevalence of bTB and an effective control program is still lacking. The multifaceted impacts and substantial economic losses render addressing bTB a daunting, but highly important challenge. In this review, we summarise all the freely available literature on *M. bovis* infection from Pakistan using Google scholar and PubMed databases. A total of 40 animal studies were identified using search terms: “bovine tuberculosis in Pakistan, bTB, Pakistan, *Mycobacterium bovis* in Pakistan, *M. bovis* in Pakistan”; while seven human studies were identified using the terms: zoonotic tuberculosis in Pakistan’, ‘*M. bovis* in humans Pakistan’, ‘zTB in TB patients in Pakistan”. We have summarized all these studies to identify critical risk factors involved in transmission of bTB among animals and humans. Despite lack of comprehensive and geographically representative studies, the literature suggests a varying prevalence of bTB in animals, ranging from as low as 2% to as high as 19%. Regarding zTB prevalence in humans, estimates range from 1.5% to 13% in high-risk group of farm and abattoir workers, with notably higher percentages in extra-pulmonary TB cases. The review also addresses the challenges that Pakistan faces in formulating an effective policy for the control and eradication of bTB. We conclude with one-health based recommendations as a way forward for controlling TB caused by *M. bovis* in cattle and humans.

## Highlights

• Pakistan lacks accurate prevalence information for *M. bovis* infection in livestock (bovine tuberculosis, bTB) and its impact on humans (zoonotic tuberculosis, zTB).
• This review covers published data until 2023 on *M. bovis* infection in humans and animals from Pakistan.
• Regional prevalence reports confirm the presence of bTB in livestock species and *M. bovis* infection in human TB patients (zTB); with no measures in place for reporting and mitigation.
• The review analyzes the challenges and risk factors for bTB and zTB; and proposes One Health-based recommendations for control of *M. bovis* infection in humans and animals.

## 1 Bovine tuberculosis and its zoonotic potential

Tuberculosis is a pervasive infectious disease with a history dating back over 3 million years [1]. *Mycobacterium tuberculosis* complex (MTBC) includes pathogenic mycobacteria such as *Mycobacterium tuberculosis* (*M. tb*), *M. bovis* [2]*, M. caprae*, *M. africanum*, *M. canetti*, *M. microtti*, *M. pinnipedii*, and *M. leprae* [3]. MTBC members demonstrate a remarkably broad host range [4]. In humans, the major infections include tuberculosis (*M. tb*), leprosy *(M. leprae*), and zoonotic tuberculosis (*M. bovis*). In cattle, *M. bovis* is the etiological agent for bovine tuberculosis (bTB) [5].

Although *M. tb* primarily causes TB in humans; however, it is also occasionally isolated from diseased cattle and buffaloes in several countries of Asia and Africa [[6], [7], [8], [9], [10]]. *M. bovis* also exhibits an extensive range of hosts, encompassing domestic animals, livestock, wildlife, and humans [11]. Transmission among animals is thought to mostly occur through the respiratory route; however, vertical transmission via the digestive route from an infected mother to calf has also been reported [12,13]. This disease prompts substantial financial losses in animals worldwide. The financial losses are estimated to reach 3 billion USD each year worldwide due to loss of production, culling of animals and trade impediments [14,15].

*M. bovis* is zoonotic in nature causing zoonotic TB (zTB) in humans. The transmission of the bacteria is linked to human-animal proximity, lack of sanitation, consumption of unpasteurized milk and uncooked meat [[16], [17], [18]]. This makes it an important public health threat for low-and-middle income countries (LMICs). In Pakistan, 63% of the population lives in rural areas [19] and 62% of these people are either directly or indirectly involved with livestock. It has been reported that *M. bovis* can contribute up to 15% of human TB cases in LMICs, compared to <1% in high income countries [4,18,[20], [21], [22]]. Concurrently, Pakistan is ranked 5th among human TB high-burden countries worldwide and alone accounts for 61% of the TB burden in the WHO Eastern Mediterranean Region [23]. Each year over 500,000 new human TB cases are reported in Pakistan [23]. However, national-level data on the prevalence of bTB and the contribution of *M. bovis* to the incidence of human TB is unavailable. The symptoms and tissue lesions caused by *M. bovis* infection in humans cannot be distinguished from those caused by *M. tuberculosis* [4,24,25]. Moreover, zTB is frequently extra-pulmonary, which complicates accurate diagnosis using conventional human TB diagnostic tests; leading to misdiagnosis or missed diagnosis of zTB [26]*.* Despite its immense economic and public health importance, there are currently no surveillance or control programs in place against bTB in Pakistan. In this manuscript, we seek to fill this paucity of knowledge by providing an up-to-date overview of bTB disease status in animals and its contribution to human TB cases in Pakistan. The review in addition, highlights the challenges and proposes a way forward for control.

## 2 Current status of bTB in Pakistan

**Fig. 1:**
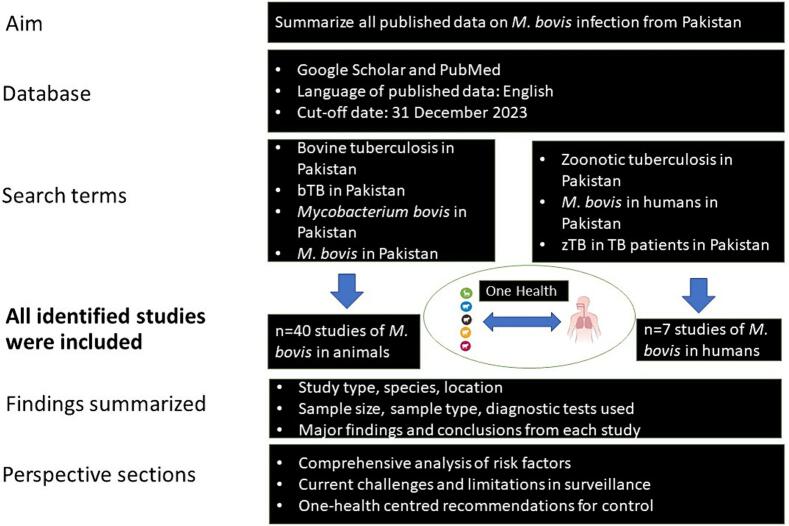
Flow chart depicting the structure of this review. Aim and the data search strategy has been described with the categories of information summarized from all the identified studies. Comprehensive analysis of risk factors, current challenges and control recommendations have also been included.

Bovine TB is endemic in Pakistan and was first reported in 1969 in district Faisalabad with a prevalence of 6.72% in dairy animals [27]. For this review, a literature search was conducted for all the studies described until 2023 on bTB in Pakistan. The strategy (presented as flow chart, Fig. 1) involved selecting all the published literature, in English language, from Google scholar and PubMed databases with the search terms: “bovine tuberculosis in Pakistan, bTB, Pakistan, *Mycobacterium bovis* in Pakistan, *M. bovis* in Pakistan”.

**Fig. 2:**
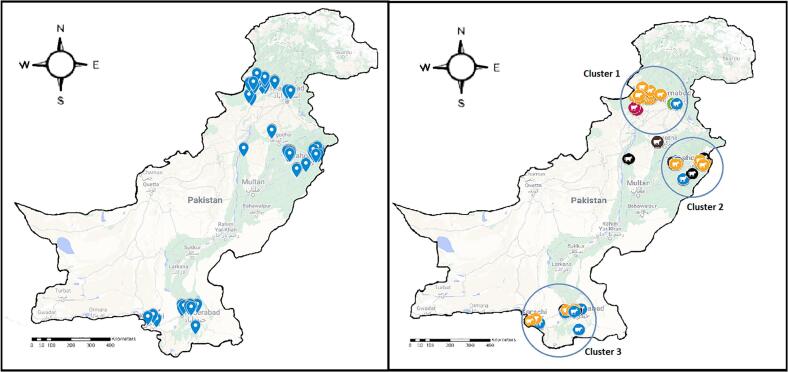
The maps demarcate the political borders of Pakistan. Left): The blue location icons represent the cities in which the 40 studies were conducted. Bovine tuberculosis prevalence studies were performed in 19 districts, with most districts targetted multiple times over the course of two decades. Right): Data points of 40 studies. Each location icon is represented by either a cow or a deer icon with different background colours. Blue colour represents studies that tested only cattle, black colour represents studies that only tested buffalos, pink colour represents studies that tested buffaloes, cattle, goat and (or) sheep, orange colour represents studies that tested both buffaloes and cattle. Green colour deer icon represents studies that tested different wildlife species. The large circles represent the three clusters of studies identified in this review paper. (For interpretation of the references to colour in this figure legend, the reader is referred to the web version of this article).

**Fig. 3:**
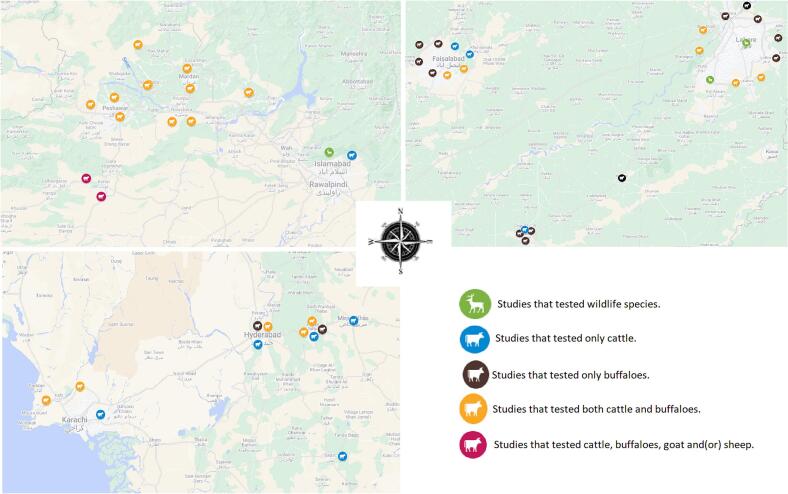
Zoomed images of the three clusters created by the 40 studies performed in Pakistan to evaluate prevalence of bovine tuberculosis. Cluster 1: Top left, Cluster 2: Top right, Cluster 3: Bottom left. Note that the location represented on the maps by each icon is that of the city and not the exact farm where the animals were tested.

The search led to the identification of 40 studies (all of them were summarized and have been presented in literature review matrix in Table 1). The studies available online were conducted over a period of two decades, even though research on bTB had been conducted earlier but was not available on online search portals. These 40 studies reported data from overlapping district locations accounting to a total of 19 districts, with most studies being conducted in Lahore (*n* = 11), followed closely by Faisalabad (*n* = 8), Tando Allahyar (*n* = 4), Peshawar (n = 4) and Okara (n = 4) (supplementary fig. 1). The 19 districts covered 56 testing sites. On mapping the study sites (Fig. 2, left), three main clusters were formed (Fig. 2, right), one in Khyber Pakhtunkhwa (KPK), the second in Northern Punjab and the third in Sindh, leaving behind Southern Punjab, Baluchistan, Gilgit Baltistan as well as Azad Jammu and Kashmir. The clustering patterns and the affiliations of the corresponding authors in those clusters suggests that the majority of studies were performed near veterinary educational institutes that had academic expertise as well as laboratory facilities to conduct the required research. The data points were further parsed based on species tested in each study (Fig. 3). The data set from Pakistan showed that out of 56 testing sites, 19 sites tested buffaloes only, 13 targeted cattle, 19 sites included cattle as well as buffaloes, two sites tested cattle, buffalo, goat (or) sheep, and three sites tested captive wild animals (including deer, chinkara, antelope, black buck, gazelle, goral, nilgai, urial and zebra) (Fig. 3).

**Table 1:** Chronological summary of bTB studies conducted in Pakistan. Geographical locations; study designs as well as major findings/conclusions have been summarized. Abbreviations: bTB, bovine tuberculosis; PPDa and PPDb, purified protein derivatives of M. avium and M. bovis; CITT, comparative intradermal tuberculin test; LJ, Lowenstein Jensen (media); SITT, single intradermal tuberculin test; ZN staining, Ziehl-Neelsen staining; AFB, acid fast bacilli.

Sr. No.	Geographical location i.e., city/district and (province)	Study design	Major findings	Reference
1.	Lahore (Punjab)	**Type of study**: cross sectional **Target animal species**: dead zoo/captive animals **Target location**: breeding parks, government and private zoos, captive wild animals **Sample size**: 185 **Sample types for testing**: 1–3 pieces of lung tissues (2 × 2 cm) preserved in buffered formalin, lung tissue samples for PCR **Diagnostic tests**: microscopic examination of HE-stained tissues; ZN staining of impression smears, PCR	• Molecular confirmation of bTB presence in wildlife • Gross lesions of bTB observed on postmortem examination in 15 dead animals (8.1%) • ZN staining from impression smears yielded positive results in 5.4% of cases • Molecular techniques, including PCR from tissue samples, detected mycobacterial DNA in 9 samples. Of these, *M. bovis* was identified in 7 samples (3.78%) and *M. tuberculosis* in 2 samples (1.1%)	[28]
2.	Mirpurkhas and Badin districts (Sindh)	**Type of study:** cross sectional **Target animal species: Cattle** **Target location: urban and peri-urban areas** **Sample size: 200** **Sample types for testing: blood, nasal discharge and milk (total 600 samples, 200 of each type)** **Diagnostic tests: SITT, *Lilli* rapid Ab test** (Lilly dale Diagnostics, England)**, ELISA, microscopy and culture (growth on LJ media supplemented with 1% sodium pyruvate; followed by niacin and urease tests** for specie differentiation**)**	• The prevalence of bTB in animals in Mirpurkhas was 5.5%, with varying prevalence detected by different tests: 4, 15,2 and 1% by SITT, rapid antibody test, culture from milk and nasal discharge, and ELISA respectively • The prevalence of bTB in animals in Badin was 7%, with varying prevalence detected by different tests: 5, 18, 3 and 2% on SITT, rapid antibody test, culture, and ELISA respectively	[29]
3.	4 public sector livestock experiment stations at Pattoki, Lahore, Bhakkar and Khushab (across Punjab)	**Type of study**: cross sectional **Target animal species**: buffaloes **Target location**: Livestock/dairy farms **Sample size**: 627 **Diagnostic tests**: CITT	• An overall prevalence of 4.3% was reported, ranging from 3.45% to 4.98% • Positive animals were detected on all four farms, indicating a 100% herd prevalence • The prevalence of tuberculosis was found to be higher in older buffaloes	[30]
4.	Bahawalnagar (Punjab)	**Type of study**: cross sectional **Target animal species**: cattle and buffalo **Target location**: Bahawalnagar **Sample size**: 340 **Sample types for testing**: blood **Diagnostic tests**: CITT and ELISA	• Out of 340 animals, 4.1% were found positive by CITT and 2.6% were confirmed positive by ELISA • Old age, herd size and purchase of animals from market were found to be risk factors • Selling of infected animals to the market was noted as significant risk factor for spread of the disease	[31]
5.	Hyderabad, Tando Allahyar (Sindh)	**Type of study**: cross sectional **Target animal species**: cattle **Target location**: Peri-urban and rural areas **Sample size**: 160 **Sample types for testing**: blood, milk, nasal discharge and feces **Diagnostic tests**: SITT, rapid bovine antibody test and culture (growth on LJ media, followed by nitrate reduction and niacin tests for specie differentiation)	• The combined prevalence of bTB in these two districts was 3.13% by SITT, 34.38% by rapid bTB antibody test and 2.4% by culture from blood, nasal, milk and fecal samples • The prevalence of bTB was found to be higher in exotic breeds compared to local breeds	[32]
6.	Khyber Pakhtunkhwa province (KPK)	**Type of study**: cross sectional **Target animal species**: cattle and buffaloes **Target location**: Peshawar, Nowshera, Charsadda, Mardan and Swabi districts **Sample size**: 2400 (1225 cattle and 1175 buffaloes) **Sample types for testing**: milk from 1608 lactating animals **Diagnostic tests**: CITT, PCR and culture (growth on Stone brink's media, followed ZN staining)	• A total of 141 animals were found to be CITT-positive, resulting in an overall prevalence of 5.88%. The species-specific prevalence in cattle was 6.45% and in buffaloes was 5.28% • **The prevalence of *M. bovis* in milk samples was found to be 3.73, 4.04 and 5.29% by ZN staining, culture and PCR respectively** • Close contact between humans and animals, consumption of raw milk, and poor sanitation were identified as major risk factors for zTB • Unrestricted animal movement due to porous borders was considered major risk factors for the spread of bTB in KPK	[33]
7.	Hyderabad, Tando Allahyar (Sindh)	**Type of study:** cross-sectional study **Target animal species:** buffaloes **Target location:** rural and peri-urban farming **Sample size:** 120 **Sample types for testing:** blood and milk **Diagnostic tests:** SITT, ELISA and culture (growth on LJ media, followed by nitrate reduction and niacin tests for specie differentiation)	• In Hyderabad, the prevalence of bTB was 1.66, 6.66 and 5% based on SITT, ELISA, andculture respectively • In Tando Allahyar, the prevalence of bTB was 6.66, 10 and 0% based on SITT, ELISA and culture respectively • The prevalence of bTB was found to be higher in older females from large herds compared to males from small herds	[34]
8.	Karachi (Sindh)	**Type of study**: cross-sectional study **Target animal species**: cattle **Target location**: two abattoirs **Sample size**: 943 (700 males and 243 females) **Sample types for testing**: blood (collected but not processed) and lungs samples (total 1170 collected including lungs 338, liver 257, lymph nodes 313, spleen 110 and intestines 152) **Diagnostic tests**: Gross examination of organs after slaughtering	• A detailed postmortem examination revealed that 95 animals/samples were positive for bTB, representing an 8.12% prevalence rate (95/1170) • Routine inspection at abattoirs only detected 15 positive cases, while 80 were incorrectly reported as negative • Inspection at abattoirs demonstrated low sensitivity, identifying only 15.79% of positive cases while misclassifying 84.21% as negative. This highlights the potential for missing TB lesions during routine abattoir inspection, with a probability of 84.21% for misdiagnosis • The frequency of lesions was highest in tracheobronchial lymph nodes (30%) followed by mediastinal lymph nodes • The prevalence of bTB was found to be lower in local breeds, possibly due to genetic characteristics that confer resistance to the disease	[35]
9.	Lahore (Punjab)	**Type of study:** cross-sectional **Target animal species:** five species of antelopes suspected of having TB **Target location:** wildlife parks and zoos **Sample size:** 100 **Sample types for testing:** blood **Diagnostic tests:** PCR and cytokine ELISA testing for *M. bovis* detection. Samples were also tested for *M. tuberculosis* to detect reverse zoonosis	• The prevalence of *M. bovis* in antelopes was found to be 30%, exceeding the prevalence of *M. tuberculosis*, which was 20%	[36]
10.	Faisalabad (Punjab)	**Type of study:** cross-sectional **Target animal species:** cattle and buffalo **Target location:** two cattle/buffalo colonies **Sample size:** 265 (133 cattle and 132 buffaloes) **Sample types for testing:** milk and nasal swabs **Diagnostic tests:** SITT, PCR and culture (media not specified)	• The overall prevalence of bTB detected using skin testing was 10.56%. While ZN staining and PCR yielded higher prevalence rates of 12.45 and 13.58%, respectively • The prevalence of bTB was higher in buffaloes (11.04%) compared to cattle (9.76%), indicating a potential breed predisposition • PCR proved to be most accurate technique and in comparison, the PPD skin test's sensitivity and specificity were 77.8% and 100%, while ZN staining sensitivity and specificity were 86.1 and 99.1%, respectively.	[37]
11.	Peshawar, Nowshera, Charsadda, Mardan and Swabi districts of Khyber Pakhtunkhwa (KPK)	**Type of study:** cross-sectional **Target animal species:** cattle and buffalo **Target location:** urban and rural areas **Sample size:** 2400 asymptomatic large animals, comprising 1225 cattle and 1175 buffaloes **Diagnostic tests:** CITT *190 dairy farmers were interviewed to gather information on animal management practices and potential risk factors for bTB	• The overall prevalence of bTB in the five districts was 5.88% • A strong association was found between bTB, animal's age, herd size, with older animals and larger herds being more likely to be infected • Only 30.1% of farmers were aware of the zoonotic nature of bTB, highlighting the need for increased education and awareness about the disease • Introducing new animals into existing herds and sheltering animals together at night were identified as additional risk factors for bTB transmission • Substandard ventilation in animal shelters was also found to contribute to the spread of bTB, as it allows for the airborne transmission of *M. bovis*	[38]
12.	Kohat - Khyber Pakhtunkhwa (KPK)	**Type of study:** cross-sectional **Target animal species:** cattle, buffalo, goat and milk shops **Target location:** 5 randomly selected union councils of district Kohat **Sample size:** 200 milk samples [cattle (62), buffaloes (64), goats (47) and milk shops (27)] **Sample types for testing:** milk **Diagnostic tests:** ZN staining followed by microscopy and PCR	• Mycobacteria were observed in 13.5% of collected milk samples on ZN staining • In milk samples from cattle, *M. bovis* prevalence was 6.4% on PCR testing • In milk samples from buffaloes, prevalence of *M. bovis* was 6.2% and prevalence of *M. tuberculosis* was 1.5% on PCR testing • In milk samples from goats, *M. bovis* prevalence was 2% and *M. tuberculosis* prevalence was 6.3% on PCR testing • Milk samples collected from shops had a higher percent positivity for *M. bovis* (7.4%) compared to the samples collected directly from animals • No milk sample was positive for *M. tuberculosis* • Older animals (over 8 years of age) were found to have a higher prevalence of bTB • Several risk factors were identified for bTB transmission, including herd size, communal grazing, watering practices, and poor sanitary conditions	[39]
13.	Karachi (Sindh)	**Type of study:** cross-sectional **Target animal species:** **Target location:** two abattoirs **Sample types for testing:** blood and tissue samples were collected from lymph node of respiratory tract, lung and liver tissue, lymph nodes from gastrointestinal tract **Diagnostic tests:** lateral flow technique, PCR, ZN staining	• 100 animals out of the 800 were suspected of having bTB • The overall prevalence of bTB was 5.87% using the lateral flow technique and 12.66% using NZ staining • Among the 100 suspected animal samples, 55 (55%) were PCR positive while 47 (47%) were ELISA positive • The prevalence of bTB increased with increasing age	[40]
14.	Karachi (Sindh)	**Type of study:** cross-sectional **Target animal species:** cattle and buffalo **Target location:** small holders dairy farms in 5 towns of Karachi **Sample size:** 1000 animals (435 cows and 565 buffaloes) **Diagnostic tests:** CITT	• Bovine TB prevalence in peri-urban Karachi was 14.4%, indicating the need for control measures • Malnourished and unhealthy animals were more susceptible to bTB infection	[41]
15.	Lahore (Punjab)	**Type of study:** cross-sectional **Target animal species:** cattle and buffalo **Target location:** 4 organized dairy farms **Sample size:** 192 animals suspected of bTB (96 cattle and 96 buffaloes) **Sample types for testing:** blood **Diagnostic tests:** SITT, ELISA and PCR	• By SITT, the prevalence of bTB was higher in cattle (11.46%) than in buffaloes (7.29%) • In buffaloes, PCR tested 85.71% animals as positive and ELISA tested 71.43% animals as positive • In cattle, PCR tested 90.91% animals as positive while ELISA tested 72.72% animals as positive • Age was identified as the most significant risk factor for bTB infection	[42]
16.	Hyderabad, Tando Allahyar (Sindh)	**Type of study:** cross-sectional **Target animal species:** cattle **Target location:** **Sample size:** 160 cattle (80 from each district) **Sample types for testing:** 160 nasal secretion and 120 milk samples **Diagnostic tests:** culture (LJ media), staining (ZN) and biochemical tests (nitrate reduction and niacin tests)	• The overall prevalence of *M. bovis* infection was 1.42%, with a higher positivity in nasal swabs (1.875%) compared to milk samples (0.833%)	[43]
17.	Peshawar – Khyber Pakhtunkhwa (KPK)	**Type of study:** cross-sectional **Target animal species:** cattle and buffalo **Target location:** small holdings and commercial farms **Sample size:** 556 animals (139 from each of four towns) were selected for testing, comprising of 368 cattle and 188 buffaloes. Of these, 143 were from smallholdings, while the remaining 443 were from commercial farms. **Diagnostic tests:** CITT	• The overall prevalence was 5.75%, with a higher prevalence in buffaloes (7.98%) compared to cattle (4.62%) • Older animals were found to be more susceptible to bTB compared to younger animals • bTB prevalence was also influenced by the origin of the animals (whether born at the same farm or purchased), their housing conditions (indoor or outdoor), and herd size	[44]
18.	Kohat - Khyber Pakhtunkhwa (KPK)	**Type of study:** cross-sectional **Target animal species:** cattle, buffalo, sheep and goats **Target location:** abattoir **Sample size:** 200 **Sample types for testing:** lungs, lymph nodes and liver **Diagnostic tests:** NZ staining and PCR	• The overall prevalence of bTB was 6.5% by PCR and 7.5% by microscopy • Prevalence varied among animal species, with the highest rates found in goats (10.6%) and sheep (6.5%), followed by cattle (5.2%) and buffaloes (4%) • Detection of bacilli was higher in lung tissues compared to lymph nodes and other tissues • The high prevalence of bTB in small ruminants could be attributed to their close proximity to infected cattle at farms	[45]
19.	Public livestock farms (Punjab)	**Type of study:** cross-sectional **Target animal species:** cattle and buffalo **Target location:** livestock farms **Sample size:** 215 **Sample types for testing:** milk and nasal swabs **Diagnostic tests:** CITT, PCR	• The overall prevalence of bTB was 24.7%, with slightly higher rates found on farm 2 (25.9%) compared to farm 1 (22.5%) when tested by CITT • The prevalence of bTB was higher in buffaloes (25.3%) compared to cattle (21.6%) when tested by CITT • PCR testing of milk and/or nasal samples from CITT-positive animals revealed 77.4% were positive for *M. bovis* • PCR testing of nasal swabs was 60% more effective in detecting positive animals compared to milk samples	[46]
20.	Faisalabad (Punjab)	**Type of study**: cross-sectional **Target animal species**: cattle and buffalo **Target location**: slaughterhouse **Sample size**: 400 **Sample types for testing**: tissues with suspected TB lesions **Diagnostic tests**: HE staining and microscopy	• The prevalence of bTB was 1.5% in buffaloes and 1% in cattle based on microscopy after HE staining • Lesions of bTB were significantly more prevalent in younger cattle less than five years of age compared to older cattle over five years of age	[47]
21.	Faisalabad (Punjab)	**Type of study:** cross-sectional **Target animal species:** crossbred cattle **Target location:** Livestock Dairy Farm, University of Agriculture Faisalabad **Sample size:** 107 **Sample types for testing:** 8 animals were positive by CITT. Six were female animals from whom milk samples were collected. Tissue samples (lung and liver) from 05 dead animals **Diagnostic tests**: CITT, ZN staining and PCR	• Prevalence of bTB was 7.47% in crossbred cattle by CCIT, which was further confirmed by microscopy and PCR	[48]
22.	Lahore (Punjab)	**Type of study:** cross-sectional **Target animal species:** cattle and buffalo **Target location:** public and private farms **Sample size:** selected 1031 (517 cattle and 514 buffaloes) animals exhibited signs of emaciation and swollen lymph nodes, suggesting potential bTB infection **Diagnostic tests:** CITT	• The overall prevalence of bTB was 2.71% based on CITT results • The study reported relatively higher prevalence estimate in buffaloes (3.3%) than cattle (2.1%)	[49]
23.	Islamabad (Islamabad Capital Territory)	**Type of study:** cross-sectional **Target animal species:** cattle **Target location:** dairy farms **Sample size:** 200 **Diagnostic tests:** CITT	• Of the 200 cattle tested, 1.5% showed a positive reaction to the PPDb antigen, while 2% showed a positive reaction to the PPDa antigen	[50]
24.	Peshawar - Khyber Pakhtunkhwa (KPK)	**Type of study:** cross-sectional **Target animal species:** cattle and buffalo **Target location:** abattoirs **Sample size:** 151 **Sample types for testing:** lung and liver tissues (302 samples were collected from an abattoir: Cattle (30 liver, 30 lung samples) and buffaloes (121 lung, 141 liver samples) **Diagnostic tests:** ZN staining and PCR	• The overall prevalence *M. bovis* infection was 14.04% in buffaloes and 13.33% in cattle, based on both ZN staining and PCR testing • The detection rate of positive samples was higher in lung tissues compared to liver	[10]
25.	Faisalabad, Okara (Punjab)	**Type of study:** cross-sectional **Target animal species:** cattle **Target location:** farm/herd **Sample size:** 521 **Diagnostic tests:** CITT	• The overall prevalence of bTB was 9.3% at the farm level, while the prevalence at the animal level was 2.3% • The prevalence of bTB increased with increasing herd size	[51]
26.	Lahore (Punjab)	**Type of study:** cross-sectional **Target animal species:** cattle **Target location:** peri-urban areas **Sample size:** 1000 **Sample types for testing:** milk **Diagnostic tests:** SITT and PCR	• The overall prevalence of bTB was 45.4% based on PCR testing of milk samples and 13.4% based on SITT • The high prevalence of bTB in milk samples poses a significant public health threat	[52]
27.	Livestock experiment stations (Punjab)	**Type of study:** cross-sectional **Target animal species:** buffalo **Target location:** livestock experiment stations **Sample size:** 965 **Diagnostic tests:** CITT	• The prevalence of bTB in animals was 11.3%, ranging from 0% to 18.8%, with 86% of farms having at least one positive animal • This rise in prevalence is largely attributed to the lack of any control policy	[53]
28.	Lahore (Punjab)	**Type of study:** cross-sectional **Target animal species:** buffalo **Target location:** buffalo/cattle colony **Sample size:** 100 **Sample types for testing:** blood **Diagnostic tests:** CITT and PCR	• The prevalence of bTB in buffaloes by CITT was 2%, with 54% of animals testing positive for *M. bovis* by PCR • PCR was found to be highly sensitive test for detection of bTB positive animals	[54]
29.	Tando Allahyar (Sindh)	**Type of study:** cross-sectional **Target animal species:** cattle and buffalo **Target location:** rural areas **Sample size:** 187 **Diagnostic tests:** SITT	• Overall prevalence on basis of SITT was 5.34%	[55]
30.	Faisalabad (Punjab)	**Type of study:** cross-sectional **Target animal species:** buffalo **Target location:** rural areas around Faisalabad (within 12 km from clock tower) **Sample size:** 1052 **Diagnostic tests**: CITT	• The overall prevalence of bTB was 2.47% • The older animals (>5 years of age) are at an increased risk of bTB infection • Animals with poor physical conditions are 2.8 times more likely to be infected with bTB than animals in good health • Animals kept in close contact with poor ventilation are also at a higher risk of bTB infection	[56]
31.	Islamabad (Islamabad Capital Territory)	**Type of study:** cross-sectional **Target animal species:** bovidae, cervidae and equidae **Target location:** zoo animals including members of Bovidae (*n* = 55), Cervidae (*n* = 31) and Equidae (*n* = 4) **Sample size:** 87 **Diagnostic tests:** CITT	• The overall prevalence was 3.3% • Two out of 55 Bovidae (3.6%); one out of 31 Cervidae (3.2%) tested positive; while no Equidae tested positive for bTB	[57]
32.	11 Livestock experiment stations (Punjab)	**Type of study:** cross-sectional **Target animal species:** cattle **Target location:** livestock experiment stations **Sample size:** 1751 **Diagnostic tests:** CITT	• The overall prevalence ranged from 2% to 19.3%, with a 100% herd prevalence, indicating the detection of at least one bTB-positive animal at each farm • Important identified risk factors included age, number of calving, total milk produced, and lactation length • Additionally, the presence of sheep and goats was identified as another significant risk factor	[58]
33.	7 Livestock experiment stations (Punjab)	**Type of study:** cross-sectional **Target animal species:** sheep and goats **Target location:** livestock experiment stations **Sample size:** 1987 goats (1472 at farms and 515 in two cities, Okara and Faisalabad) and 4987 sheep (4729 at farms and 254 in two cities, Okara and Faisalabad) **Diagnostic tests:** CITT	• The prevalence of bTB was 0.9% in sheep and 2.4% in goats • A positive bTB case was found in 100% of goat farms, while in sheep farms, positive reactors were detected in 86% of farms • The percentage of reactors was higher when small ruminants were kept with large ruminants	[59]
34.	Faisalabad, Okara (Punjab)	**Type of study:** cross-sectional **Target animal species:** buffalo **Target location:** rural areas/villages **Sample size:** 1092 (697 from Faisalabad and 395 from Okara) **Diagnostic tests:** CITT	• The overall prevalence of bTB in buffaloes by CITT was 2.6% • It was noted that the prevalence of bTB was higher when cattle were also present at buffalo farms	[60]
35.	Lahore, Faisalabad, Okara (Punjab)	**Type of study:** cross-sectional **Target animal species:** buffalo **Target location:** dairy farms **Sample size:** 395 **Diagnostic tests:** CITT	• Overall prevalence was found to be 2.22% • Old age and number of calving were important risk factors of bTB	[61]
36.	Lahore (Punjab)	**Type of study:** cross-sectional **Target animal species:** buffalo **Target location:** cattle colonies and dairy farms in the peri-urban areas **Sample size:** 31 **Sample types for testing:** milk **Diagnostic tests:** CITT and PCR	• *M. bovis* was detected in 29% of the animals through PCR of milk samples, while the prevalence of bTB through CITT was 9.6%	[62]
37.	Livestock experiment station, Khushab (Punjab)	**Type of study:** cross-sectional **Target animal species:** buffalo **Target location:** livestock experiment station **Sample size:** 159 **Sample types for testing:** milk and feces **Diagnostic tests:** CITT, culture, ZN staining and isolation (growth on LJ and Stone brink's media, followed by niacin, nitrate reduction, catalase and urease tests for specie differentiation)	• The prevalence by CITT was 10.06% • Out of 16 skin test-positive animals, 56.25% were also positive by bacterial culture • Buffaloes aged >8 years had a higher prevalence of 68.75% • Milk was identified as the most significant risk factor for zoonotic TB infection in humans	[63]
38.	Lahore, Okara, Faisalabad (Punjab)	**Type of study:** cross-sectional **Target animal species:** buffalo **Target location:** livestock experiment stations **Sample size:** 2526 **Diagnostic tests:** CITT	• The overall prevalence was 12.72%, ranging from 8.52% to 19.4% • Important risk factors include poor feeding, sanitation, wildlife interactions, and other management practices	[64]
39.	Livestock experiment stations (Punjab)	**Type of study:** cross-sectional **Target animal species:** buffalo **Target location:** livestock experiment stations **Sample size:** 328 (165 from farm 1 and 163 from farm 2) **Diagnostic tests:** CITT	• The overall prevalence by CITT was 5.48%, ranging from 2.45% to 8.58% • Identified potential risk factors include age, number of animals, frequent visitors, contact with wild animals, backyard poultry, and management practices	[65]
40.	Lahore (Punjab)	**Type of study:** cross-sectional **Target animal species:** cattle and buffalo **Target location:** cattle colonies **Sample size:**1000 **Sample types for testing:** milk, nasal discharge and feces **Diagnostic tests:** CITT	• Overall prevalence of bTB was 7.3% • Prevalence of bTB was 6.91% in buffaloes and 8.64% in cattle • Mycobacterium AFB were identified through microscopy after isolation from 16 milk samples (28.07%) and 7 nasal samples (12.28%) from buffaloes with positive skin test results. Additionally, 4 milk samples (25%) and 2 nasal samples (12.5%) from cattle exhibited the presence of AFB	[66]

Table 1 summarizes all studies conducted on bTB in Pakistan along with their study designs, sampling/approaches used and major conclusions drawn by the authors. Most studies adopt a similar approach, reporting prevalence estimates using a variety of diagnostic approaches. While we understand the funding constraints for the local researchers, many studies lack a proper study design and comparison to gold-standard mycobacterial culture for confirmation. Nonetheless, these studies suggest that the prevalence of bTB is <10% in many areas, while exceeding 10% in other locations, with estimated rates varying from 2% to 19.3%. Differences in bTB prevalence arise from diverse factors, including geographical variations influenced by farm management conditions [67]. Natural susceptibility levels also vary among species and breeds, as highlighted in two studies [46,68]. Additionally, the variation in bTB prevalence is influenced by topography, climate, weather patterns, and the presence of diverse animal species including wildlife. Notably, these estimations often stem from limited studies rather than comprehensive, geographically representative investigations [69]. The listed studies also suggest that the prevalence of bTB is increasing at well-established government production farms, which is alarming [46,53]. Different risk factors identified in studies conducted in Pakistan include the age of the animal, close animal sheltering, number of calving, origin of animals (brought in by purchasing from other locations) and herd size.

Studies conducted in Pakistan have also shown that bTB is present in captive wild animals. A study conducted recently proved that bTB is present in zoo animals with prevalence range of 3.78% to 8.1% depending on diagnostic tool employed [28]. Similarly, results of a 2019 study on captive antelopes, which belong to the Bovidae family, demonstrated an *M. bovis* infection incidence of around 30%, suggesting a high affinity for bTB in these animals [36]. In 2015, a study conducted on zoo animals belonging to bovidae, cervidae and equidae family indicated an overall prevalence estimate of 3.3% [57]. These studies suggest that bTB is present in captive wildlife as well as zoo animals, which poses immense challenge of spread of bTB not only to animals but also people working at these facilities and visiting as tourists.

The studies reported in the current review used a variety of TB diagnostic tools such as the single intra-dermal tuberculin test (SITT; *n* = 7), comparative intra-dermal tuberculin test (CITT; *n* = 23), PCR (*n* = 14), rapid bovine antibody test (*n* = 3), ELISA (*n* = 5), Ziehl-Neelsen (ZN) staining (*n* = 10), culture (n = 7), and gross examination of visceral organs especially lungs and liver (n = 1) (supplementary table 1). Of the 40 studies, 20 studies employed only one form of test while the remaining 19 studies used a combination of tests to determine the status of bTB in animals. On further exploration of the publications, only seven studies used mycobacterial culture to confirm the presence of live bacteria (supplementary table 1). In addition, molecular tests were only employed in few research studies where sophisticated laboratories were accessible [33]. Therefore, variable prevalence of bTB was reported by different studies: making it difficult to ascertain the true picture of bTB in Pakistan. The situation is further complicated by the fact that most studies utilized convenience sampling that can only provide an estimate rather than the true prevalence of the disease. Nonetheless, the studies provide a starting point for the government agencies to initiate an integrated surveillance study to ascertain the true prevalence of bTB. Overall, it is not possible using the currently available data to accurately assess the impact of bTB on either the bovine or human population (next sections) in Pakistan.

## 3 Status of zoonotic tuberculosis due to *M. bovis* infection in Pakistan

Research articles that determined causative contribution of *M. bovis* in human TB cases were searched using Google scholar and PubMed with the following inputs: ‘zoonotic tuberculosis in Pakistan’, ‘*M. bovis* in humans Pakistan’, ‘zTB in TB patients in Pakistan’. The information is summarized in Table 2. A total of seven studies were identified from the search that spanned over a decade starting from 2012. No studies performed prior to that were available online. Furthermore, these studies have been conducted in five cities that represent only three of the five provinces of Pakistan. These provinces include Baluchistan [Quetta (*n* = 1)], KPK [Peshawar (*n* = 4)] and Punjab [Gujranwala and Hafizabad (n = 1), Lahore (n = 1)]. The studies report variable percentages of zTB ranging from 1.5% to 13% in high risk groups such as farm workers and abattoir workers [70]. The percentage of zTB was also found to be drastically different between pulmonary and extra-pulmonary human TB cases [71]. Ali et al.*,* [71] reported that 5 out of 244 pulmonary TB patients (2.5%) and 19 out of 91 extra-pulmonary TB patients (20.9%) tested positive for *M. bovis*. The results should have concerned the local TB reporting authorities, since one-fifth of all extra-pulmonary cases in the study were zoonotic that required immediate intervention by the public health authorities. Yet the public health domain to-date remains unconcerned about zTB and the impact it would have on the goal of having a TB-free Pakistan by 2035. With sustained transmission of TB from animals to humans, this goal seems unrealistic.

**Table 2:** List of studies conducted in Pakistan to access contribution of *M. bovis* in human TB cases (zTB). The abbreviations: LJ, Lowenstein Jensen (media); PCR, Polymerase chain reaction; ZN staining, Ziehl-Neelsen staining; zTB, zoonotic tuberculosis*.*

Sr. No.	Geographical area	Study design	Major findings	Reference
1.	Quetta (Balochistan)	**Target population:** suspected TB patients (presented with chronic signs such as cough, night sweat, fever, weight and appetite loss) **Sample size:** 200 **Sample collected:** sputum **Tests conducted:** fluorescent microscopy and PCR	• Fluorescent microscopy detected 31 positive cases, while PCR detected 60 and 2 positive cases for *M. tb* and *M. bovis* respectively. • PCR shown to be more sensitive and effective for specie differentiation compared to fluorescent microscopy	[72]
2.	Peshawar - Khyber Pakhtunkhwa (KPK)	**Target population:** livestock farm workers, abattoir workers, butchers, veterinarians and veterinary assistants **Sample size:** 390 **Sample collected:** sputum samples were collected: human TB patients from different hospitals (100); livestock workers (200); abattoir workers (23); butchers (35); veterinarians (10); and veterinary assistants (22) **Tests conducted:** ZN staining, culture (LJ and stone brink's media followed by morphological characterization) and PCR	• *M. bovis* was detected in 2 out of 100 TB patients (2%), 3 out of 200 farm workers (1.5%), and 3 out of 23 abattoir workers (13%) through PCR. 2 patients positive for *M. bovis* were also confirmed by culture. • No butcher, veterinarian and veterinary assistant detected positive for *M. bovis* • Slaughterhouse labor are more affected due to a lack of precautionary measures and inadequate protection (no availability of PPE) • There is a lack of knowledge about bTB/*M. bovis*, its transmission, zoonotic potential, and public health significance among farm as well as abattoir workers	[70,73]
3.	Peshawar - Khyber Pakhtunkhwa (KPK)	**Target population:** clinically diagnosed TB patients (before start of any medication) and school children **Sample size:** 300 **Sample collected:** sputum **Tests conducted:** culture (on LJ and stone brink's media) and PCR	• Out of 300 TB patients 4% (*n* = 12) tested positive for *M. bovis* and 96% (*n* = 288) were confirmed for *M. tb* through PCR. However, 274 samples showed growth on LJ media showing presence of *M. tb* isolates, 13 samples showed growth on stone brink media indicating presence of *M. bovis*. Interestingly, 5 samples showed growth on both media suggesting mixed infection • All samples from school children tested negative. • Except for one, all (11) *M. bovis* isolates were resistant to pyrazinamide	[74]
4.	Peshawar - Khyber Pakhtunkhwa (KPK)	**Target population:** occupationally exposed individuals **Sample size:** 103 samples were collected from occupationally exposed individuals with clinical signs: abattoir workers (16), butchers (29), livestock farmers (50), veterinarians (3), and veterinary assistants (5) **Sample collected:** sputum **Tests conducted:** PCR	• 25% (*n* = 4) of abattoir workers and 2% (n = 1) of farmers tested positive for *M. bovis* • Abattoir workers face a higher risk due to direct exposure to numerous cases without PPE • Occupationally exposed groups lack knowledge and formal training • The duration of work for an abattoir worker was identified as a risk factor for infection	[75]
5.	Peshawar - Khyber Pakhtunkhwa (KPK)	**Target population:** TB patients admitted in hospitals **Sample size:** 100 **Sample collected:** sputum **Tests conducted:** culture (LJ and stone brink media followed by nitrate reduction test), ZN staining and PCR tests	• 2% (*n* = 2) of the TB patients tested positive for *M. bovis* while 98% were confirmed positive for *M. tuberculosis* using the PCR test • Out of 100, 96 samples shown growth on LJ media showing presence of *M. tb* and 4 showed growth on stone brink media indicating presence of *M. bovis*	[76]
6.	Gujranwala and Hafizabad districts (Punjab)	**Target population:** suspected TB patients **Sample size:** 335 **Sample collected:** sputum, pus, fluid from lymph nodes, peritoneal effusion and pleural effusion while samples could not be collected from 11 patients **Tests conducted:** ZN staining and PCR	• Out of 324 TB positive patients 244 had pulmonary TB while 91 were cases of extra pulmonary TB • Out of 324 samples that tested positive on ZN staining, 7.4% (*n* = 24) were positive for *M. bovis* on PCR • Out of 244 pulmonary TB patients only 5 were detected positive for *M. bovis* while out of 91 extra pulmonary TB patients 16 detected positive for *M. bovis* • Prevalence of *M. bovis* in cases of extra-pulmonary TB is very high compared to pulmonary TB	[71]
7.	Lahore (Punjab)	**Target population:** suspected TB patients **Sample size:** 100 **Sample collected:** 200 samples (100 blood and 100 sputum samples) **Tests conducted:** culture (LJ and stone brink media; followed by niacin accumulation and nitrate reduction tests), ZN staining and PCR	• Out of 100 sputum samples, 37% (*n* = 37) were positive for *M. tb* and 5% (*n* = 5) were positive for *M. bovis* by PCR • From 100 blood samples, 4% (n = 4) were found *M. bovis* positive by PCR • On culture, 11 sputum samples were found positive for *M. tb* • The authors reported a sensitivity of 100% and specificity of 70.79% for Duplex PCR test with a positive and negative predictive value of 29.73 and 100% respectively	[77]

## 4 Risks factors for zTB in Pakistan

There are many factors which exacerbate the spread of *M. bovis* to humans and *M. tb* from human to animals (reverse zoonosis) in LMICs [78]. In their studies, Desta et al.*,* [79] and Kouengoua et al.*,* [80] identified several direct and indirect factors that can influence the spread of tuberculosis between animals and humans. These include demographics like age and gender, along with dietary habits such as consuming unpasteurized milk and meat. Close contact with livestock also plays a role, whether through shared living spaces, common water sources, or direct interactions. The presence of tuberculosis within a household, either human or animal, further increases the risk. Practices like spitting indoors and using manure in animal feed can also contribute to transmission. Furthermore, poor ventilation in homes and a lack of awareness about TB transmission modes can hinder control efforts.

In Pakistan, people in rural areas often have traditional livestock housing systems with close contact between humans and animals, thereby increasing the risk of zTB transmission from infected animals [81]. Similarly, sale and purchase of infected animals in the market is found to be a significant risk factor [31]. Co-sleeping in poorly ventilated areas with animals and consumption of unpasteurized milk and dairy products in rural Pakistani settings is quite common [74,81]. In fact, most people in rural areas who are already at high risk due to close contact with infected animals prefer drinking raw milk, which further increases the risk of *M. bovis* transmission [56,82]. Yet there is no mandatory pasteurization law in Pakistan to prevent transmission of *M. bovis* through contaminated milk. Currently only 6% of total milk produced is pasteurized [83]. In 2018, Punjab Food Department enacted a law called ‘Minimum Pasteurization Law’ in Punjab, which to date has not been implemented in the country [84].

Another important risk factor for transmission of *M. bovis* to humans is through handling of contaminated carcasses at abattoirs [85]. Unfortunately, the awareness level in slaughterhouse workers and animal owners regarding the risk of zoonosis is very low in Pakistan: as demonstrated by a study conducted at abattoirs in Karachi reporting that only 15% workers and herdsmen knew about bTB and only 30% workers were taking some precautions such as wearing of personal protection equipment and washing their hands [81]. These risk factors have been observed globally: in Mexico 11.8% of sputum samples from cattle farm workers were positive for *M. bovis* [86], while in Argentina, 65% of *M. bovis*-infected patients were found to have occupational exposure [87].

The role of National Tuberculosis Control Program (NTP) in humans becomes important under current conditions. Yet the NTP in Pakistan has not even acknowledged the existence of zTB and its contribution to the TB burden in a largely agrarian country [88]. The absence of legislation mandating pasteurization, coupled with indoor animal housing practices and insufficient awareness within susceptible populations, has afforded *M. bovis* unchecked transmission to humans, thereby posing enormous health challenges to public health.

## 5 Challenges in control of bTB and zTB in Pakistan

Developed nations (global north) have successful programs that have reduced infections in animals to <1% at the herd level. Unfortunately, many LMICs lack similar efforts since it requires knowledge of data on prevalence and associated risk factors in each country. Absence of systematic surveillance and incomplete data on disease incidence and prevalence results in lack of attention and resources from policymakers [24,82,[89], [90], [91]]. Furthermore, compromised field performance of the available diagnostic tests for bTB i.e., low sensitivity and specificity, is also a challenge [[92], [93], [94]]. Bovine TB prevalence is primarily concentrated in specific regions where poverty intersects with high population density, a predictable outcome for an illness that thrives in areas with limited resources to prevent bTB transmission [95,96].

Pakistan is among the LMICs that have yet to develop a surveillance program to effectively determine the prevalence of bTB in animals [30,75]. The veterinary education establishments such as University of Veterinary Animal Science Lahore and University of Agriculture Faisalabad have conducted small scale studies to determine the prevalence of bTB (Table 1). Yet, the Ministry of National Food Security and Research of Pakistan does not have programs to conduct active surveillance of bTB even though they have the required infrastructure in place that was previously developed during the eradication of Rinderpest and maintained for the current control efforts being targeted towards Foot-and-Mouth Disease (FMD) control.

Majority of the bTB studies have been conducted around metropolitan cities like Karachi and Lahore; where animals have been removed from city centres and gathered in the peri-urban areas in dense dairy colony structures. This facilitates the transport of milk directly to consumers within a short frame of time. The forced clustering of animals in small areas has likely led to ideal conditions for the increased transmission of *M. bovis* in animals*.* Indeed, two studies have demonstrated significantly high prevalence of bTB in both animals and milk samples [41,52]. This is worrisome since the dairy animals that are brought to the colonies get infected and at the end of the lactation-cycle are either sent to the slaughterhouse or back to their original villages, facilitating transmission of bacteria to different parts of the country.

A significant challenge in control of bTB in animals is the lack of knowledge regarding the dynamics of *M. bovis* infection in wildlife. Wildlife hosts such as white-tailed deer in Michigan (USA), Eurasian badgers in the UK, brush-tailed possums in New Zealand etc. are considered complicating factors in control of bTB in developed countries [97]. In Pakistan, no study till date has assessed the prevalence of bTB in wildlife species while only a handful studies have reported prevalence of bTB in captive wildlife (Table 1). Lack of bTB data from both captive and wildlife species also makes it difficult to assess their potential role (if any) in transmission of bTB to farm animals.

Another important challenge is the non-existence of government policies for the control of bTB. Policies such as those related to animal movement across borders, insufficient funding for culling, lack of quarantine facilities, shortages of meat inspectors and veterinarians in slaughterhouses; all contribute to a hostile environment for bTB control [98]. Pakistan has porous borders with Afghanistan and Iran leading to frequent cross-border animal and human movement, which is very serious risk factor for spread of infectious diseases like bTB [38,70]. This, combined with lack of a surveillance program and a coordinated effort between veterinary research and educational establishments in the country to conduct a thorough research are major hurdles in initiating control of bTB.

So far, the challenges described above were specific to control of bTB in animals in Pakistan. Yet the zoonotic nature of this disease with ∼ 10% contribution to human TB cases in LMICs [22], makes it imperative that we highlight the challenges faced in preventing transmission of the bacteria from infected animals to the human population, since the clinical presentation of tuberculosis caused by *M. bovis* and *M. tuberculosis* is indistinguishable. The African region has the highest cases of zTB (72700), followed by Southeast Asian region (46700); yet they lack sufficient resources, laboratory infrastructure as well as technical expertise for differentiating *M. bovis* from *M. tuberculosis* at essentially all healthcare levels [[99], [100], [101]]**.** Additionally, there is absence of training and lack of knowledge among occupationally exposed groups about the zoonotic potential and spread of *M. bovis* infection to humans [75]. According to studies conducted to evaluate knowledge and awareness of different exposed high risk groups, it was found that none of the abattoir workers received formal training and majority of them do not practice any precaution such as wearing PPE and washing of their hands [74,75]. In another study, it was established that abattoir workers are more affected by zTB due to direct contact and continuous exposure [74]. The condition is worsened because *M. bovis* inherently exhibits resistance to pyrazinamide [102]. In a Peshawar-based study, 12 patients were diagnosed with *M. bovis* infection and 11 of those patients' demonstrated resistance to pyrazinamide treatment. This underscores a significant challenge posed by antimicrobial resistance associated with zTB [70].

It is crucial that bTB in animals is controlled to prevent its transmission to humans. On the human health side, *M. bovis* is neither considered a risk factor for human TB, nor are there any guidelines for its control in the newly formed “National Guidelines for the Control of Tuberculosis in Pakistan-2019” [88]. This is irrespective of the numerous studies having reported the impact of *M. bovis* on human TB (zTB) cases in veterinarians, livestock workers, butchers, abattoir workers and other high risk groups Table 2: List of studies conducted in Pakistan to access contribution of *M. bovis* in human TB cases (zTB) [70]. Lack of concentrated efforts by animal and human health departments so far has led to a precarious situation that needs immediate attention.

## 6 Future perspectives for bTB and zTB control in Pakistan

The way forward for Pakistan to control bTB is to carry out comprehensive surveillance to determine incidence and prevalence of *M. bovis* infection in cattle and other hosts, along with risk assessment of zoonosis and reverse zoonosis. The data regarding prevalence of bTB and share of zTB in human TB should be compiled. This will be helpful in efficient allocation and management of resources [69] because the lack of data is the main hurdle in seeking policymakers' attention and provision of funding to fight the problem [103,104].

There is a need to identify and address research gaps in zoonotic and bovine TB to determine the true burden of TB caused by *M. bovis* and to unravel the epidemiology of disease at the human, animal, and environmental interface [26]. This can be achieved through meaningful collaboration between local researchers and experts from developed world and international organizations working on TB control. This will also offer capacity building opportunity for the local researchers, enabling them to better design future research studies. Additionally, in endemic countries like Pakistan, epidemiological studies to determine which animal species, both domestic and wild, serve as hosts or reservoirs for *M. bovis* can be very informative. This process can identify areas of high prevalence, enabling the allocation of additional funds and efforts to those specific regions [105,106].

Routine surveillance of bTB in live animals through skin testing and blood tests can play vital role in control of disease. Animal owners should be incentivized for screening their animals for bTB and maintenance of disease free herds [104]. Additionally, inspection of the animals prior to slaughtering and detailed post-mortem inspection (following the protocols implemented in UK and the EU) should be ensured to stop entry of meat of any infected animal into the food chain [107,108]. This should be accompanied by tracing back to the herd to which the diseased animal belonged [101].

The effective control of zoonotic diseases is only possible by adopting multi-sectorial, one health approach [109,110]. There is need to design a national control program with integrated ‘One Health’ approach through collaboration between public health and animal health departments with aim to develop control measures while considering human, animals, wildlife and environmental fronts [104,111]. The success of the TB control program in humans in the USA is commendable, owing to collaborative efforts from states, businesses, and the federal government to control and eradicate the disease [104]. Pakistan can establish a similar program through collaboration between the federal and provincial governments on both animal and human health sector. The program should emphasize surveillance to determine the prevalence of bTB and identify risk factors. Integrated and collective control efforts are imperative: including awareness among masses, necessary legislation, and its implementation along with uplifting socioeconomic status of the people.

The surveillance campaign to detect cases of human TB due to *M. bovis* should be strengthened by improved laboratory facilities, including polymerase chain reaction (PCR) and gene sequencing after culturing the isolates from patients [77].

Pasteurization and food safety laws should be introduced with strict implementation by adopting WHO food safety measures to break the cycle of transmission of *M. bovis* to the masses. This will require a public awareness campaign to educate people about the importance of pasteurizing milk and other dairy products [26]. The control strategies can be made successful by educating the masses, especially the farmers about the economic and public health significance.

Field veterinarians and para-veterinary staff should be aware of the mode of transmission, range of hosts, clinical signs, and diagnosis of the disease [29,74]. There is also need for implementation of standard operating procedures (SOPs), creating a national database, training of technical and non-technical staff for ante-mortem and post mortem examination along with educating animal handlers to break the cycle of zTB transmission [75,112].

## 7 Conclusion

The absence of comprehensive data for bTB and zTB in Pakistan has resulted in failure to develop a structured control and eradication program. Recognizing this critical gap, it is imperative to develop an effective policy that prioritizes the control and eradication of bTB and zTB. A One-Health approach is advocated, calling for collaboration among various departments such as livestock and dairy development, ministry of national health services, public health, agriculture, and food security to collectively achieve targeted goals. Furthermore, there is a pressing need for active surveillance to accurately determine the prevalence of *M. bovis* and its contribution to the human TB burden. This data is crucial not only for understanding the disease's national status but also for identifying pertinent risk factors. Such insights are integral in the formulation of a comprehensive control program against the disease and for efficient resource allocation. In conclusion, representative data is pivotal for addressing the substantial challenge posed by bTB to both livestock and human population. The establishment of a robust control program, informed by accurate prevalence data and a One-Health approach, is imperative for mitigating the impact of this infectious disease in Pakistan.

## CRediT authorship contribution statement

**Zahid Fareed:** Conceptualization, Data curation, Formal analysis, Investigation, Methodology, Writing – original draft, Writing – review & editing. **Aysha Rana:** Conceptualization, Data curation, Methodology, Writing – original draft. **Syeda Anum Hadi:** Data curation, Formal analysis, Methodology, Software, Writing – original draft. **Annemieke Geluk:** Supervision, Writing – review & editing. **Jayne C. Hope:** Supervision, Writing – review & editing. **Hamza Khalid:** Conceptualization, Formal analysis, Project administration, Resources, Supervision, Validation, Writing – original draft, Writing – review & editing.

## Declaration of competing interest

The authors declare that they have no known competing financial interests or personal relationships that could have appeared to influence the work reported in this paper.

## Data Availability

No data was used for the research described in the article.

## Appendix A Supplementary data

Details of the districts where bTB studies included in this review were performed and the tests used by each study to detect bTB. Image 1
