# Supplementary material for: A one health-focused literature review on bovine and zoonotic tuberculosis in Pakistan from the past two decades: challenges and way forward for control
Source: One Health. 2024 May 23;18:100763. doi: 10.1016/j.onehlt.2024.100763 (PMC11153871; doi:10.1016/j.onehlt.2024.100763)
Supplement: Supplementary file 1 — Details of the districts where bTB studies included in this review were performed and the tests used by each study to detect bTB. [file mmc1.docx]

**Supplementary Materials**

**A One health-focused literature review on bovine and zoonotic tuberculosis in Pakistan from the past two decades: challenges and way forward for control.**

Zahid Fareed ^a^, Aysha Rana ^a^, Syeda Anum Hadi ^b^, Annemieke Geluk ^c^, Jayne C. Hope ^d^, Hamza Khalid ^c,d,e*^

^a^ *Veterinary Research Institute, Lahore, Punjab, Pakistan.*

^b^ *Consultant-Technical Coordinator, Health Security Partners, Islamabad, Pakistan.*

^c^ *Department of Infectious Diseases, Leiden University Medical Center, Leiden, The Netherlands.*

^d^ *Division of Immunology, The Roslin Institute, University of Edinburgh, EH25 9RG, UK.*

^e^ *Center for Inflammation Research, The Queen’s Medical Research Institute, Edinburgh BioQuarter, 47 Little France Crescent, Edinburgh EH16 4TJ, UK*

*Corresponding author: [h.khalid@lumc.nl](mailto:h.khalid@lumc.nl)

Supplementary Table 1: Types of diagnostic tests used in the selected studies for diagnosing bTB in animals from 2003-2023. The abbreviations: CITT, comparative intradermal tuberculin test; SITT, single intradermal tuberculin test; ZN staining, Ziehl-Neelsen staining; AFB, acid fast bacilli

| **Study References** | **CITT** | **SITT** | **ZN staining/AFB** | **Rapid bovine antibody test** | **ELISA** | **PCR** | **Culture** | **Gross examination** | **Types of tests used per study** |
| --- | --- | --- | --- | --- | --- | --- | --- | --- | --- |
| [1-17] | **✓** |  |  |  |  |  |  |  | 1 |
| [18] |  |  |  |  |  |  |  | **✓** | 1 |
| [19] |  | **✓** |  |  |  |  |  |  | 1 |
| [20] |  |  | **✓** |  |  |  |  |  | 1 |
| [21] |  |  | **✓** |  |  | **✓** |  |  | 2 |
| [22] |  |  |  |  | **✓** | **✓** |  |  | 2 |
| [23] |  |  | **✓** |  |  | **✓** |  |  | 2 |
| [24] |  |  | **✓** |  |  |  | **✓** |  | 2 |
| [25] |  |  | **✓** |  |  | **✓** |  |  | 2 |
| [26] | **✓** |  |  |  |  | **✓** |  |  | 2 |
| [27] |  |  | **✓** |  |  | **✓** |  |  | 2 |
| [28] |  | **✓** |  |  |  | **✓** |  |  | 2 |
| [29] | **✓** |  |  |  |  | **✓** |  |  | 2 |
| [30] | **✓** |  |  |  |  | **✓** |  |  | 2 |
| [31] |  | **✓** |  | **✓** |  |  | **✓** |  | 3 |
| [32] | **✓** |  |  |  |  | **✓** | **✓** |  | 3 |
| [33] |  | **✓** |  |  | **✓** |  | **✓** |  | 3 |
| [34] |  | **✓** |  |  |  | **✓** | **✓** |  | 3 |
| [35] |  | **✓** |  |  | **✓** | **✓** |  |  | 3 |
| [36] | **✓** |  | **✓** |  |  | **✓** |  |  | 3 |
| [37] | **✓** |  | **✓** |  |  |  | **✓** |  | 3 |
| [38] |  |  | **✓** | **✓** | **✓** | **✓** |  |  | 4 |
| [39] |  | **✓** | **✓** | **✓** | **✓** |  | **✓** |  | 5 |
| [40] | **✓** |  |  |  | **✓** |  |  |  |  |
| Total of each test | **24** | **7** | **10** | **3** | **6** | **14** | **7** | **1** |  |

*Supplementary figure 1. The graph represents the 19 different districts where the studies included in the current review were performed.*

**References:**

1. Ehtisham-ul-Haque, S., et al., *Monitoring the Health Status and Herd-Level Risk Factors of Tuberculosis in Water Buffalo (Bubalus bubalis) Dairy Farms in Pakistan.* Pakistan Veterinary Journal, 2021. **41**(4).

2. Ullah, A., et al., *Bovine Tuberculosis (bTB): Prevalence and Associated Risk Factors in Large Ruminants in the Central Zone of Khyber Pakhtunkhwa, Pakistan.* Pakistan Journal of Zoology, 2019. **51**(1).

3. Memon, M., et al., *Prevalence and risk factor analysis of bovine tuberculosis in bovine population in Karachi.* Pakistan. J. Anim. Health. Prod, 2017. **5**(2): p. 44-49.

4. Khattak, I., et al., *Risk factors associated with Mycobacterium bovis skin positivity in cattle and buffalo in Peshawar, Pakistan.* Trop Anim Health Prod, 2016. **48**(3): p. 479-85.

5. Shaukat Ali, R.A., Muhammad Younus, Gulbeena Saleem, Qamar Un Nisa and Beenish Zahid, *Comparative trends of bovine tuberculosis in cattle and buffalo population around Lahore, Pakistan.* European Journal of Environmental Ecology, 2014. **1**: p. 7-11.

6. A. Azam, Q.S., U. Younas, A. Husna, N.Ullah, Q. Ali and S. Akhter, *Hematological studies among bovine tuberculosis suspected herds of cattle in suburb of Islamabad, Pakistan.* Wayamba Journal of Animal Science, 2014.

7. Javed, M.T., et al., *Brief communication (Original). Certain risk factors associated with positive SCCIT test for tuberculosis in cattle at two cities in Pakistan.* Asian Biomedicine, 2013. **7**(2): p. 267-274.

8. Javed, M.T., et al., *Analysis of some of the epidemiological risk factors affecting the prevalence of tuberculosis in buffalo at seven livestock farms in Punjab Pakistan.* Asian Biomedicine, 2012. **6**(1): p. 35-42.

9. Arshad, M., et al., *Epidemiological studies on tuberculosis in buffalo population in villages around Faisalabad.* Journal of Animal and Plant Sciences, 2012. **22**(3): p. 246-249.

10. Shahid, A., et al., *Prevalence of bovine tuberculosis in zoo animals in Pakistan.* Iranian Journal of Veterinary Research, 2012. **13**(1): p. 58-63.

11. Javed, M.T., et al., *Risk factors identified associated with tuberculosis in cattle at 11 livestock experiment stations of Punjab Pakistan.* Acta tropica, 2011. **117**(2): p. 109-113.

12. Javed, M.T., et al., *Percentage of reactor animals to single comparative cervical intradermal tuberculin (SCCIT) in small ruminants in Punjab Pakistan.* Acta Tropica, 2010. **113**(1): p. 88-91.

13. Javed, M.T., et al., *Risk factors associated with the presence of positive reactions in the SCCIT test in water buffalo around two cities in Punjab, Pakistan.* Acta tropica, 2010. **115**(3): p. 242-247.

14. Javed, M.T., A. Farooqi, and H. Ullah, *Epidemiological basis of bovine tuberculosis in buffaloes.* Pakistan Journal of Zoology, 2009. **9**: p. 417-420.

15. Khan, I. and A. Khan, *Prevalence and risk factors of bovine tuberculosis in Nili Ravi buffaloes in the Punjab, Pakistan.* Italian Journal of Animal Science, 2007. **6**(sup2): p. 817-820.

16. Javed, M.T., et al., *A study on tuberculosis in buffaloes: some epidemiological aspects, along with haematological and serum protein changes.* Veterinarski arhiv, 2006. **76**(3): p. 193-206.

17. Jalil, H.D., P; Suleman A et al., *Bovine tuberculosis in Dairy Animals at Lahore, Threat to the Public Health. Available online.* 2003.

18. Bhutto, A.L., et al., *Prevalence and pathological lesions of bovine tuberculosis assessment through routine procedures of meat inspection in infected cattle in Karachi metropolitan corporation abattoirs.* Pure and Applied Biology, 2019. **8**(3): p. 1909-1918.

19. Qazi, I.H., et al., *Prevalence of Bovine Tuberculosis in Rural Areas of District Tando Allahy ar.*

20. Waqas, A. and M. Javed, *K. Ashfaque, Mehwish Q (2015) An Abat-toir Based Study on Brucellosis, Bovine Tuberculosis and Paratubercu-losis in Buffaloes and Cattle at Faisalabad, Pakistan.* Int J Vet Health Sci Res, 2015. **3**(1): p. 34-38.

21. Akhtar, K., et al., *Molecular identification and infection pathology of Mycobacterium spp. in captive wild animals in Pakistan.* The Journal of Infection in Developing Countries, 2023. **17**(08): p. 1107-1113.

22. Akhtar, R., et al., *Use of molecular probes for presumptive diagnosis of tuberculosis associated with Mycobacterium tuberculosis and Mycobacterium bovis infection in antelopes in Pakistan.* Bovis, 2019. **53**(52.6): p. 470.

23. Basit, A., et al., *Occurrence and risk factors associated with Mycobacterium tuberculosis and Mycobacterium bovis in milk samples from North East of Pakistan.* Tuberculosis, 2018.

24. Leghari, A., et al., *Isolation of Mycobacterium bovis from milk and nasal discharge samples of cattle from Hyderabad and Tando Allahyar districts.* J. Anim. Health Prod, 2016. **4**(4): p. 105-110.

25. Basit, A., et al., *Isolation and identification of Mycobacterium bovis and Mycobacterium tuberculosis from animal tissues by conventional and molecular method.* Indian Journal of Animal Research, 2015. **49**(5): p. 687-693.

26. Akhtar, F., et al., *The use of PCR technique in the identification of Mycobacterium species responsible for bovine tuberculosis in cattle and buffaloes in Pakistan.* Tropical animal health and production, 2015. **47**: p. 1169-1175.

27. Khan, J., et al., *Prevalence of tuberculosis in buffalo and cattle.* J Pure Appl Microbiol, 2014. **8**: p. 721-726.

28. Tipu, M.Y., et al., *A cross sectional study of Mycobacterium bovis in dairy cattle in and around Lahore city, Pakistan.* Pakistan Journal of Zoology, 2012. **44**(2).

29. Khan, A., et al., *Detection of Mycobacterium bovis in buffaloes blood through polymerase chain reaction (PCR) and tuberculin test.* JAPS, Journal of Animal and Plant Sciences, 2012. **22**(3 Supplement): p. 237-241.

30. Mumtaz, N., et al., *Reliability of PCR for detection of bovine tuberculosis in Pakistan.* Pakistan Journal of Zoology, 2008. **40**(5).

31. Leghari, A., et al., *Prevalence and Risk Factors Associated with Bovine Tuberculosis in Cattle in Hyderabad and Tando Allahyar Districts, Sindh, Pakistan.* Pakistan Journal of Zoology, 2020. **52**(1).

32. Ullah, A., et al., *Bovine tuberculosis (bTB)-isolation and species-specific identification of Mycobacterium bovis from bovine raw milk in Pakistan.* Sarhad J. Agric, 2020. **36**(2): p. 489-498.

33. Malhi, K.K., et al., *Prevalence of bovine tuberculosis in buffaloes in Hyderabad and Tando Allahyar districts of Sindh, Pakistan.* Indian Journal of Animal Research, 2020. **54**(1): p. 101-105.

34. Aslam, M.S., et al., *Bacterial and PCR based diagnosis of naturally occurring bovine tuberculosis in cattle and buffaloes.* Pakistan Journal of Agricultural Sciences, 2019. **56**(2).

35. A. Tariq, A.A., Y. Tipu, M. Ahmad, R. Sultan, A. Anjum, *A preliminary study on prevalence of bovine tuberculosis in cattle and buffalo in outskirts of Lahore, Pakistan.* Wayamba Journal of Animal Science, 2017(1482427224).

36. Mahmood, F., et al., *Molecular based epidemiology of bovine pulmonary tuberculosis–a mortal foe.* Pakistan Veterinary Journal, 2014. **34**(2): p. 185-188.

37. Imtiaz A. Khan, A.K., A. Mubarak and S. Ali, *Factors affecting prevalence of bovine tuberculosis in Nili Ravi buffaloes.* Pakistan Veterinary Journal, 2008: p. 155-158.

38. M. R. Memon, A.L.B., M. I. Memon , P. Khatri and J. A. Baloch, *Prevalence of bovine tuberculosis in slaughtering animals at selected municipal slaughter houses: its impact on public health.* Pakistan Journal of Agriculture, Agricultural Engineering and Veterinary Sciences, 2018: p. 168-175.

39. Mazari, M.Q., et al., *Prevalence and Risk Factors of Bovine Tuberculosis in Cattle and Dairy Farm Workers in Mirpurkhas and Badin Districts of Sindh, Pakistan.* Pakistan Journal of Zoology, 2022. **54**(3): p. 1115.

40. Zahoor, M.Y., *A cross-sectional study of bovine tuberculosis and its associated zoonotic risk factors in district Bahawalnagar, Punjab, Pakistan.* Board of Reviewing Editors, 2021. **51**: p. 192-193.
